# Alzheimer-related genes show accelerated evolution

**DOI:** 10.1038/s41380-020-0680-1

**Published:** 2020-03-13

**Authors:** Anne Nitsche, Christian Arnold, Uwe Ueberham, Kristin Reiche, Jörg Fallmann, Jörg Hackermüller, Friedemann Horn, Peter F. Stadler, Thomas Arendt

**Affiliations:** 1 Bioinformatics Group, Department of Computer Science, University Leipzig, Härtelstraße 16-18, D-04107 Leipzig, Germany; 2 Department of Diagnostics, Fraunhofer Institute for Cell Therapy and Immunology – IZI, Perlickstraße 1, D-04103 Leipzig, Germany; 3 Computational EvoDevo, Department of Computer Science, University Leipzig, Härtelstraße 16-18, D-04107 Leipzig, Germany; 4 Paul Flechsig Institute of Brain Research, Liebigstraße 19, 04103 Leipzig, Germany; 5 Bioinformatics Unit, Department of Diagnostics, Fraunhofer Institute for Cell Therapy and Immunology – IZI, Perlickstraße 1, D-04103 Leipzig, Germany; 6 Young Investigators Group Bioinformatics and Transcriptomics, Department Molecular Systems Biology, Helmholtz Centre for Environmental Research – UFZ, Leipzig, Germany; 7 Department of Computer Science, University Leipzig, Leipzig, Germany; 8 Interdisciplinary Center for Bioinformatics, University Leipzig, Härtelstraße 16-18, D-04107 Leipzig, Germany; 9 Max Planck Institute for Mathematics in the Sciences, Inselstraße 22, D-04103 Leipzig, Germany; 10 Department of Theoretical Chemistry, University of Vienna, Währingerstraße 17, A-1090 Wien, Austria; 11 Center for non-coding RNA in Technology and Health, University of Copenhagen, Grønnegårdsvej 3, DK-1870 Frederiksberg C, Denmark; 12 Santa Fe Institute, 1399 Hyde Park Rd., Santa Fe, NM 87501 USA

**Keywords:** Psychiatric disorders, Neuroscience

## Abstract

Alzheimerʼs disease (AD) is a neurodegenerative disorder of unknown cause with complex genetic and environmental traits. While AD is extremely prevalent in human elderly, it hardly occurs in non-primate mammals and even non-human-primates develop only an incomplete form of the disease. This specificity of AD to human clearly implies a phylogenetic aspect. Still, the evolutionary dimension of AD pathomechanism remains difficult to prove and has not been established so far. To analyze the evolutionary age and dynamics of AD-associated-genes, we established the AD-associated genome-wide RNA-profile comprising both protein-coding and non-protein-coding transcripts. We than applied a systematic analysis on the conservation of splice-sites as a measure of gene-structure based on multiple alignments across vertebrates of homologs of AD-associated-genes. Here, we show that nearly all AD-associated-genes are evolutionarily old and did not originate later in evolution than not-AD-associated-genes. However, the gene-structures of loci, that exhibit AD-associated changes in their expression, evolve faster than the genome at large. While protein-coding-loci exhibit an enhanced rate of small changes in gene structure, non-coding loci show even much larger changes. The accelerated evolution of AD-associated-genes indicates a more rapid functional adaptation of these genes. In particular AD-associated non-coding-genes play an important, as yet largely unexplored, role in AD. This phylogenetic trait indicates that recent adaptive evolution of human brain is causally involved in basic principles of neurodegeneration. It highlights the necessity for a paradigmatic change of our disease-concepts and to reconsider the appropriateness of current animal-models to develop disease-modifying strategies that can be translated to human.

## Introduction

Alzheimer’s disease (AD) is an age-related chronic neurodegenerative disorder of unknown cause. Its etiology likely involves complex genetic and environmental traits [1]. Neuropathologically, it is characterized by extracellular neuritic plaques mainly consisting of aggregated Aß-peptide derived from the much larger amyloid precursor protein (APP) and intracellular neurofibrillary tangles composed of fibrillar aggregates of the microtubule-associated protein tau in a hyperphosphorylated form. The discovery that autosomal dominant forms of AD are attributable to mutated genes coding for APP or one of the presenilins, proteins that contain the catalytic activity of gamma-secretase, releasing Aß from APP, prompted continuous efforts to establish ‘transgenic mice models of AD’ for therapeutic research. Over the last 20 years, these transgenic mice have been state-of-the-art tools to develop preventive or therapeutic strategies against AD. Still, the tremendous success to ameliorate amyloid pathology and cognitive dysfunction in these mice models that have been observed in more than 300 reports could not be translated into effective therapies for AD patients [2–4]. While several attempts have been made to explain this apparent discrepancy [2], overall it highlights the necessity to reconsider our approaches to define the molecular pathology of AD and the appropriateness of model systems to validate therapeutic concepts.

AD is a human-specific disorder. While deposits of Aß have been described in birds, fish, and various mammalian species, neurofibrillary tangles are found almost exclusively in humans, and even non-human primate only develop an incomplete form of the disease [5–7]. This exclusivity of AD to human brain clearly implies a phylogenetic aspect of the disease and most likely indicates that adaptive changes of cerebral structure and function that have occurred during human evolution may have rendered the human brain sensitive to AD [8]. Clear cut phylogenetic traits of the AD pathomechanism might have far reaching consequences with respect to our approaches of disease prevention and therapy including defining appropriate animal model systems. Still, this evolutionary dimension remains difficult to prove and has not been established unequivocally so far.

Major phenotypic changes that have occurred in the course of human evolution, especially those between humans and chimpanzees, are suggested to mainly result from an increase in gene expression and are, thus, reflected at the transcriptomic level [9–11]. Genes whose expression have increased in human brain are mainly related to growth and differentiation [12] and frequently are involved in transcriptional regulation and RNA processing [9, 10]. While major evolutionary changes might have occurred at the transcriptomic level, they appear to be particularly pronounced for non-coding RNAs (ncRNAs). As shown by analyses of sequenced genomes of a large variety of species, the relative amount of non-coding sequence increases consistently with complexity [13]. Thus, ncRNAs, most likely constitute a critical layer of gene regulation in complex organisms that have expanded during evolution [14]. They have been conceptualized as a “complexity multipliers” in the human brain, allowing the ~30 000 protein coding genes to code for ~10^6^ synapses [15].

To test the hypothesis that brain evolution critically contributes towards the AD pathomechanism, here, we established the AD-associated genome-wide RNA profile comprising both protein-coding (cRNA) and non-protein-coding (ncRNA) transcripts and applied a systematic analysis on the conservation of splice sites as a measure of the evolution of gene structure.

## Methods

### Patients and healthy controls

Brain tissue of 19 AD patients and 21 healthy controls dying without any history of neurological or psychiatric illness was used. The diagnosis of AD was made on the basis of both clinical and neuropathological evidence according to the criteria of the International Working Group (IWG) for New Research Criteria for the diagnosis of AD in the revision of 2014 (IWG-2) [16], the NIA-AA diagnostic criteria in the revision of 2011 [17] and the NIA-AA guidelines for the neuropathological assessment of AD [18, 19]. Only cases with typical AD according to IWG-2 criteria were included. All cases underwent neuropsychological assessment within the last 6 months prior to their death. Clinical Dementia Rating (CDR) scale scoring was based on neuropsychological testing (CERAD) [20], MMSE [21] and rating scales [22]. All cases were neuropathologically assessed for NFT stage, Aß/amyloid plaque score and for neuritic plaque score according to [18]. NFTs and Aß/amyloid plaques were detected by immunecytochemical labeling of phospho-tau (anti-human PHF-tau monoclonal antibody AT8; Thermo Scientific) and Aß (beta amyloid monoclonal antibody, 6E10; BioLegend), respectively. Case recruitment, autopsy and data handling have been performed in accordance with the ethical standards as laid down in the 1964 Declaration of Helsinki and its later amendments as well as with the convention of the Council of Europe on Human Rights and Biomedicine and had been approved by the responsible Ethics Committee of Leipzig University (GZ 01GI9999-01GI0299; Approval # 282–02). Informed consent was obtained from all subjects or their legal representatives.

### Genome-wide RNA profile and analysis of splice site conservation

**Fig. 1:**
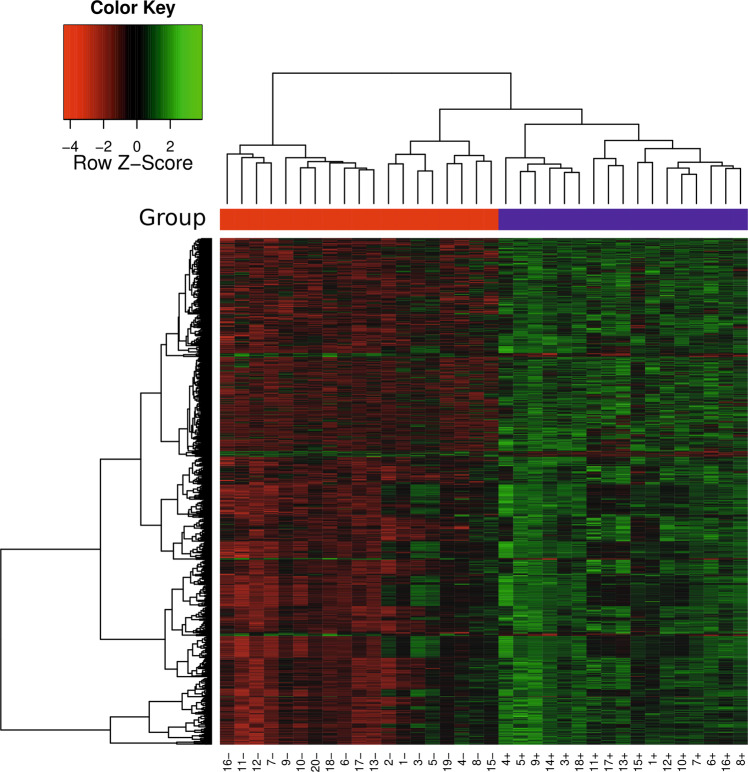
Heatmap of differentially expressed probes: Heatmaps (with dendrogram) of differentially expressed probes, based on normalized expression values for *q* = 0.1. Each row shows one differentially expressed probe and each column a particular array (i.e., patient). For each array, the patient group (blue: AD, red: control) is depicted.

The AD-associated genome-wide RNA profile comprising both protein-coding (cRNA) and non-protein-coding (ncRNA) transcripts was established combining whole genome tiling arrays with a custom array approach. To this end, we designed a custom array comprising 931 898 probes derived from Agilents Whole Human Genome Oligo array, long non-coding RNA (lncRNA) probes extracted from public databases, computationally predicted loci of structured RNAs, and lncRNA probes experimentally identified by transcriptome-wide expression variation studies based on the Affymetrix Human Tiling 1.0 array comparing AD patients with control samples (Supplementary methods and results; Supplementary Tables S1, S2). This custom array allows us to not only rely on available annotation for transcripts, but extends the search space by computationally predicted loci and expression studies. Applying this custom array to 17 AD patients and 19 control samples, we identified a differential expression of 154 multi-exonic cRNAs with a total of 4 162 splice sites and 141 multi-exonic lncRNAs with a total of 1 297 splice sites (Supplementary methods and results, Section 2.2). In Fig. 1 we show a heatmap representation of differentially expressed probes highlighting the commonalities within and differences between the AD and control patient groups. While Supplementary Table S3 provides an overlap of identified AD-associated genes with known AD-associated genes, differentially expressed coding and non-coding genes are listed in Supplementary Tables S4, S5.

To establish a measure of the evolution of gene structure, we applied a systematic analysis on the conservation of splice sites based on multiple alignments [23] across 18 vertebrates (Supplementary Tables S6, S7) of homologs of AD-associated protein-coding and non-coding genes (Supplementary methods and results, Section 1.7).

## Results

In order to compare the conservation of genes at a structural level we consider two levels of resolution. We can ask whether the gene is present or absent in another species as an entity, i.e., without considering details of the intron-exon structure. This amounts to pinpointing its evolutionary origin. More stringently we may ask whether the exact intron-exon structure, and hence the human layout, is present in a non-human primate. Genes that are present but have changed their structure likely have undergone more dramatic functional changes than genes whose structure has been exactly preserved. Protein-coding genes and non-protein-coding genes were independently investigated for their conservation (Supplementary Fig. S1).

**Fig. 2:**
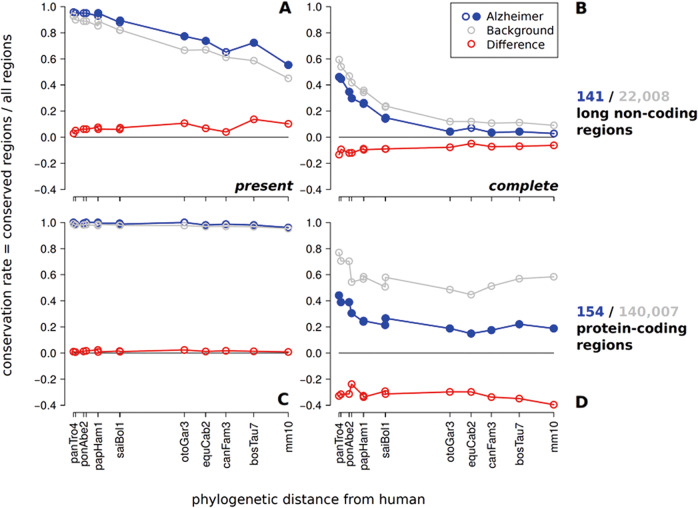
Conservation rates of human AD-associated non-protein-coding (**a**, **b**) and protein-coding (**c**, **d**) regions comparing present and completely conserved gene structure. On the horizontal axis mammalian species are indicated (denoted by the UCSC abbreviations) at their phylogenetic distance from human. Distinct data points are connected by lines to guide the eye. Variations in assembly and alignment quality cause some non-monotonicity in the curves, the overall decrease of conservation with phylogenetic distance is nevertheless clearly visible. Statistical significance of differences is computed independently for each species. Filled circles indicate *p* < 0.05. The fraction of detectable conserved AD-associated non-coding transcripts (141) is marginally higher than the conservation of the background set non-coding transcripts (22,008) if only presence/absence of a transcript is considered (A/C). In contrast, if conservation of the entire gene structure is considered (B/D), AD-associated genes are significantly less conserved than the control. This is true for both lncRNAs (**b**) and protein-coding genes (**d**, 154 AD-associated, 140,007 control). We also show intermediate levels of gene structure conservation in the Supplementary Figs. S2, S3. Additional controls against possible confounding effects e.g. of alignment quality in Supplementary Figs. S4, S5 demonstrate that the trends found here are robust.

Nearly all AD-associated protein-coding genes are evolutionarily old (Fig. 2c). There were no differences in conservation rate when comparing presence of AD-associated and all protein-coding genes. This means that AD-associated protein-coding genes did not originate later in evolution than other protein-coding genes. In line with previous reports [14], lncRNAs are much less well conserved and many have emerged in the course of mammalian evolution. The fraction of conserved lncRNAs thus decreases rapidly with evolutionary time (Fig. 2a, b; Supplementary Figs. S2, S3). As for protein-coding sequences (Fig. 2c, d) we did not observe a significantly younger origin of AD-associated genes.

While there is no recognizable difference in the evolutionary age of origin between AD-associated genes compared to the transcriptome as a whole (Fig. 2c), we observed significant, albeit more subtle differences in the evolution of AD-associated and lncRNAs in general, concerning the changes in gene structure. The conservation rate of AD-associated non-coding genes decreases significantly (*p* < 0.05) below background level (Fig. 2a, b) not only for distantly related mammals but even primates, when complete conservation of gene structure is considered. In other words, the fraction of transcripts that have the entirety of their splice sites conserved is smaller amongst AD-associated ncRNAs than amongst non-coding genes at large. AD-associated ncRNAs hence show an accelerated evolution of their gene structure. This is indicative of a more rapid functional adaptation of AD-associated non-coding genes.

Although protein-coding genes are much better conserved than lncRNAs we observed the same increase of splice site turnover in AD. In fact, the relative effect is even stronger compared to non-protein-coding loci (~30–40% vs. ~5–15% difference, shown as red lines in Fig. 2b, d, respectively). Since the same fraction of transcripts is present, while the conservation rate decreases with a more stringent conservation level of gene structure, we conclude that splice sites are systematically less conserved in human AD-associated regions compared to the typical behavior of the transcriptome.

While protein-coding loci exhibit an enhanced rate of small changes in their gene structure, we observe large changes in lncRNAs, again with a significantly enhanced rate in the AD-associated ncRNAs. The observed trends are robust as controls against possible confounding effects, e.g., of alignment quality demonstrate (Supplementary Figs. S4, S5). This suggests that in particular AD-associated non-coding genes play an important, as yet largely unexplored, role in the AD pathomechanisms.

## Discussion

Genome-wide studies that systematically analyze the evolutionary age of protein-coding and non-protein-coding AD-associated genes have not been performed previously. While major evolutionary changes might have occurred at the transcriptomic level, they appear to be particularly pronounced for lncRNAs [23, 24]. As shown by analyses of sequenced genomes of a large variety of species, the relative amount of non-coding sequence increases consistently with complexity [13]. Thus, lncRNAs, most likely constitute a critical layer of gene regulation in complex organisms that has expanded during evolution [14]. However, the evolutionary histories of lncRNAs have been notoriously hard to study due to their usually low level of sequence conservation. This not only hampers comprehensive homology-based annotation efforts but also makes it nearly impossible to obtain the high fidelity sequence alignments that are required for in depth studies into their evolution. Alternatively, the conservation of gene structure and particularly the conservation of splice sites may also be used to establish homology of lncRNAs [23]. Splice sites therefore leave “phylogenetic footprints”, and conserved patterns of splice sites may be used to predict novel transcripts from multiple genome alignments [25, 26]. Although lncRNAs are clearly ancient components of vertebrate genomes, they exhibit a rapid turnover of their intron/exon structures [23] that may be indicative of functional adaptation.

While the disease-relevance of lncRNAs is increasingly recognized, previous systematic gene expression profiling studies in AD nevertheless focused predominantly on protein-coding genes. Consequently, so far, only a few individual AD-associated ncRNAs have been identified and functionally characterized [27].

We have shown here that gene structures of AD-associated loci evolve faster than the genome at large, while there is no evidence that AD-associated genes originated particularly late in evolution. In order to capture the evolution of lncRNAs, we focused on gene structure, i.e., the conservation of splice sites because this approach makes it possible to separate the evolution of the transcripts from other selective constraints such as regulatory DNA elements that may affect sequence conservation [23]. Changes in gene structure can be expected to have in general larger functional effects than point mutations. The enhanced rate of gene structure evolution in AD-related genes hints a relation of AD to recent adaptive evolution, presumably in relation to the rapid evolution of the human brain. Importantly, replacing the background set by only genes expressed in brain did not affect the conclusions (Supplementary methods and results, Section 2.3).

The most pronounced cortical expansion during hominid evolution occurs in the association cortex [28], i.e., brain areas that show most significant differences in gene expression between the human and non-human primate brain [10, 29], and are affected in AD most early and most constantly [30]. Evolutionary expansion of the neocortex, and in particular phylogenetic shaping of association areas, is associated with a developmental deceleration and an extended period of high neuronal plasticity into adulthood [12]. The presence of these neurons which remain structurally immature throughout their lifespans might provide the prerequisite both for the human adaption to the “cognitive niche” and for a high vulnerability towards factors that lead to the development of AD [31].

Our data support the concept that neurodegeneration in AD is a result of the evolutionary legacies that have occurred during the course of evolution of the human brain. This might explain why the ability to develop AD-type pathology has emerged only in lower primates and further complemented along the line leading to monkeys, great apes and humans [5], and why early symptoms of AD typically affect mental abilities that have been acquired only during very recent hominid evolution and as such are specific to human [32].

Our results also suggest that the mode of action of a number of life style factors that have been identified as disease modifying factors such as physical activity, body fat, systolic blood pressure, stress, alcohol and tobacco consumption might be viewed against the background of genetic and environmental conditions that have resulted in the evolutionary emergence of humans in their current biological and socio-cultural form. In addition, genetic risk factors such as for instance ApoE, one of the strongest genes contributing to AD, should be considered against the background of adaptive mechanism occuring during hominid brain evolution [33].

This phylogenetic trait of AD highlights the necessity for a paradigmatic change of our concepts of the disease. Non-primate mammals, including rodents, are not very likely reliable models able to adequately mimic critical pathogenetic events of AD and to identify potential therapeutic targets. We thus need to reconsider our approaches to define the molecular pathology of AD and the appropriateness of current animal model systems to develop disease-modifying strategies.

## Supplementary information

Supplementary Methods and Results

Supplementary Figures S1-S5

Supplementary Tables S1-S3

Supplementary Table S4

Supplementary Table S5

Supplementary Table S6

Supplementary Table S7

## Data Availability

Tiling Array and Custom Array data are available at Gene Expression Omnibus (GSE138261).
