# Supplementary material for: Alzheimer-related genes show accelerated evolution
Source: Mol Psychiatry. 2020 Mar 13;26(10):5790–6. doi: 10.1038/s41380-020-0680-1 (PMC8758480; doi:10.1038/s41380-020-0680-1)
Supplement: Supplementary file 1 — Supplementary Methods and Results [file 41380_2020_680_MOESM1_ESM.docx]

**Supplement – Alzheimer-related genes show accelerated evolution**

Anne Nitsche^1^, Christian Arnold^1-3^**, Uwe Ueberham^4^**, Kristin Reiche^5^**, Jörg Fallmann^1*^, Jörg Hackermüller^6,7^*, Friedemann Horn^2^*, Peter F. Stadler^1,2,8−12^*, Thomas Arendt^4^*

^1^ Bioinformatics Group, Department of Computer Science, University Leipzig, Härtelstraße 16-18, D-04107 Leipzig, Germany

^2^ Department of Diagnostics, Fraunhofer Institute for Cell Therapy and Immunology – IZI, Perlickstraße 1, D-04103 Leipzig, Germany

^3^ Computational EvoDevo, Department of Computer Science, University Leipzig, Härtelstraße 16-18, D-04107 Leipzig, Germany

^4^ Paul Flechsig Institute of Brain Research, Liebigstraße 19, 04103 Leipzig, Germany

^5^ Bioinformatics Unit, Department of Diagnostics, Fraunhofer Institute for Cell Therapy and Immunology – IZI, Perlickstraße 1, D-04103 Leipzig, Germany

^6^ Young Investigators Group Bioinformatics and Transcriptomics, Department Molecular Systems Biology, Helmholtz Centre for Environmental Research – UFZ, Leipzig, Germany

^7^ Department of Computer Science, University Leipzig, Leipzig, Germany

^8^ Interdisciplinary Center for Bioinformatics, University Leipzig, Härtelstraße 16-18, D-04107 Leipzig, Germany

^9^ Max Planck Institute for Mathematics in the Sciences, Inselstraße 22, D-04103 Leipzig, Germany

^10^ Department of Theoretical Chemistry, University of Vienna, Währingerstraße 17, A-1090 Wien, Austria

^11^ Center for non-coding RNA in Technology and Health, University of Copenhagen, Grønnegårdsvej 3, DK-1870 Frederiksberg C, Denmark

^12^ Santa Fe Institute, 1399 Hyde Park Rd., Santa Fe, NM 87501, USA

*Corresponding authors

**Contributed equally

**1. Supplementary Methods**

**1.1. RNA isolation**

RNA was isolated by Trizol™ method (Invitrogen, Karlsruhe, Germany). 100mg deeply frozen human brain tissue (temporal cortex) was homogenized in the presence of 1ml Trizol in a glass-Teflon™ homogenizer. The homogenate was transferred to a microtube and after adding chloroform, samples were centrifuged at 15 000 g (4 °C) for 15min and the supernatant transferred to a fresh tube. Samples were mixed with equal amounts of isopropanol and centrifuged at 12 000 g (4 °C) for 15min to precipitate the RNA. After washing, the pellet was air-dried and dissolved in water. RNA quality was assessed by denaturing formaldehyde agarose gel electrophoresis, by spectrophotometry (scanning at 220−320 nm) and by analysis using Agilent 2100 bioanalyzer. Only samples with RIN > 5 were further processed. The RNA concentration was estimated spectrophotometrically by absorbance at 260 nm, concentration was adjusted to 1mg/ml and RNA was stored at −80 °C until use.

**1.2. Whole genome tiling arrays**

Equal masses of total RNA derived from three patient and three control samples, respectively, were pooled. The Affymetrix Human Whole Genome Tiling Array 1.0 Set consisting of 14 arrays was used according to the manufacturer’s instructions, except that separate labeling reactions were used for each array starting from 10 μg pooled total RNA.

We used the TileShuffle algorithm described in ^1^ to determine expressed and differentially expressed genomic intervals. Affymetrix Human Whole Genome Tiling Array 1.0 Set raw signal intensities were mapped to human genome version NCBI36 using Affymetrix BPMAP files.^2^ Expressed segments were detected with the TileShuffle parameter settings: window size = 200, the window score was defined as the arithmetic mean trimmed by the maximal and minimal values over signal intensities of all probes in a window, number of permutations = 10 000 and number of GC classes = 4. All windows with an adjusted p < 0.05 according to Benjamini and Hochberg ^3^ were defined to be significantly expressed. DE-TARs are differentially expressed TileShuffle intervals with adjusted p < 0.05 (window size = 200, the window score was defined as the logfold-change discarding all probes with converse behavior as observed for the relevant significantly expressed windows, number of permutations = 100 000 and number of GC classes = 1). Finally, the genome coordinates of all significantly expressed and all significantly differentially expressed segments were lifted over to GRCh37 (hg19) using. ^4^

**1.3. Design of the Alzheimer Custom Microarray**

Genomic intervals that were found expressed in the tiling array approach in AD or control were combined with regions we found differentially expressed in tiling array experiments on p53 induction, STAT3-signaling, cell cylce phases, and macroRNAs called STAiRs, described in, ^5^ a list of manually curated AD-associated genes from literature, and other sources of annotated or predicted ncRNAs for probe design: Known lncRNAs retrieved from public databases – NONCODE,^6^ lncRNAdb,^7^ fRNAdb,^8^ RNAdb,^9^ HInvDB,^10^ Gencode v4,^11^ RefSeq,^12^ from literature - lncRNAs originating from actively transcribed genes,^13^ chromatin-associated RNAs,^14^ snoRNAs from the snoBoard database,^15^ intronic RNAs identified in ^16^, and genomic intervals with RNA secondary structure under stabilizing selection (RNAz,^17^ Evofold.^18^ Since natural antisense transcripts appear to regulate transcription and translation of neighboring genes (e.g.,^19^ we designed probes antisense to protein-coding genes (Gencode v4). MRNAs were represented by Agilent’s 026652 catalog probe set, which is based on human RefSeq mRNA sequence. Also, we designed probes for all protein-coding genes found additionally in Gencode v4.

Custom microarray probe design is a non-trivial task for pervasively transcribed genomes. The CEM-designer pipeline ^20^ was therefore used to facilitate (i) the collection and generation of a set of unified target sequences and (ii) the selection of a set of sensitive and specific probes that represent the target sequences best while meeting space constraints of the array. Target sequences shorter than 60 bp and duplicate target sequences (i.e., identical start and end positions) were discarded. Parts of non-coding annotations that overlapped coding sequences were removed to enable a clear separation between probes interrogating non-coding and coding transcripts.

Probe design was performed using Agilent’s eArray platform, using standard parameters for expression arrays, in particular 60 bp probes and the base composition methodology, which aims at equally distributing probes across the target sequence. Probe uniqueness was checked against human genome assembly version hg19, rigorously discarding non-uniquely mapping probes using BLAT with options that maximize sensitivity (-stepSize=5 -repMatch=1000000-fine -minIdentity=90). This design strategy ensured that probes were unique both on the DNA and RNA level (according to human genome version GRCh37/hg19 and all known RefSeq transcripts, respectively).

The number of probes per target sequence was set in dependence to target length. Target sequences were represented by exactly one probe if the length was 60 ≤ l < 300, three probes if the length was 300 ≤ l < 600, and five probes if the length was 600 ≤ l < 1 000. Target sequences longer than 1 000 bp were split into intervals of 60 bp overlapping 1 000 bp chunks to ensure that probes may also be designed in the vicinity of the split positions. Each subsequence was then treated as an individual region subject to the design strategy as described above. For target sequences with an unknown reading strand (e.g., sequences originating from the various tiling array experiments, ncRNA predictions, and chromatin-associated ncRNAs), we designed probes for both strands.

Overall, the Alzheimer Custom Microarray contains 931 898 probes of which 905 197 are custom probes. A summary of the genomic distribution of probes is shown in Supplementary table S1.

**1.4. Processing of the Alzheimer Custom Microarray**

Total RNA quality was checked using Agilent’s 2100 Bioanalyzer and only samples with a RIN ≥ 5.0 were retained for microarray analysis. For 19 patient and 21 control samples 1 μg of total RNA was labeled using the Quick Amp Labeling Kit (Agilent, Waldbronn, Germany), according to the manufacturer’s instructions with the adaptation of using 120 pmol of a random N6 − T7 primer (Metabion, Planegg, Germany) instead of a polyT-T7 primer. cRNA quantity was checked using a NanoDrop ND-1000 UV-VIS Spectrophotometer, as enlisted in the manufacturer’s instructions. 1.65 μg of labeled cRNA was used for hybridization following manufacturer’s instructions. After hybridization the arrays were washed according to the manual and scanned using the Agilent G2565CA Microarray Scanner System with Agilent Scan Control Software (Version A851) following settings for scanning: Profile: AgilentG3 GX 1Color; Channels Green; Scan Region: Agilent HD (61 × 21.6mm); Resolution 3 μm double pass; Tiff: 20 bit; Green PMT Gain: 100%. Result tables were extracted after grid placement using Agilent Feature Extraction Software (Version 11.5.1.1)

**1.5. Identification of Differentially Expressed Probes**

Differential expression analysis was performed using R and the Bioconductor package Limma.^21^ Quality control of arrays was performed by checking distribution of “bright corner”, “dark corner” probes, and relative spike-in concentration versus normalized signal. The controls confirmed high quality of the results of 17 patients with Alzheimer disease and 19 controls, that were included in the downstream analysis. Initially, independent filtering was performed, removing probes (i) with signal intensity above the background in less than one third of all arrays and (ii) exhibiting an interquartile range of log2 signal intensity across all samples of less than 1. Background expression was defined by the mean intensity plus three times the standard deviation of negative control spots (Agilent’s 3xSLv spots). 113 047 out of 931 898 probes were retained after filtering. Signal intensities were quantile normalized ^22^ but not background corrected, due to the low background intensities of Agilent arrays.

Differential expression between AD and control samples was determined using a linear model that includes age because on average, individuals from the AD group were older than controls (81 and 65 years, respectively):

*E[Xi] = α × AD + β × Age + ϵ* (1)

where *E[Xi]* is the expected expression of probe *i, ϵ* an error term, *α* the coefficient modeling the impact of AD on the expression variance of probe *i*, and *β* the coefficient modeling the influence of the patient’s age. The linear model was fitted using the R package limma and reliable variance estimates were obtained by Empirical Bayes moderated t-statistics. False discovery rate was controlled by a modified Benjamini-Hochberg procedure that incorporates an estimated proportion of the null p-values ^23^ to compute q-values using the *fdrtool* R package.^24,25^

With q < 0.2, a comparably relaxed cutoff for controlling the false discovery rate of individual probes was chosen, because individual probes were subsequently aggregated for each annotated item, as described below. Probes with meeting this cutoff and uniquely mapping to the genome defined the set *s_diff_* that we used for all subsequent analyses.

**1.6. Identification of Differentially Expressed Loci**

Subsequently, we identified differentially expressed loci aggregating differentially expressed probes. The rationale behind this step was to identify the set of genes that show particularly trustworthy signs of differential expression. We argue that the differential expression of an individual probe may not be a sufficient criterion for the corresponding gene to be considered differentially expressed. For example, consider the following case for a particular gene g for which one differentially expressed probe p_diff_ mapping to g has been identified. Among all probes that map to g, p_diff_ may be a false positive, and all other probes do not show signs of differential expression. Thus, further incorporating g may not be useful because other genes show much stronger and homogeneous signals with respect to differential expressions of probes.

Therefore, we considered loci as differentially expressed if a significant fraction of probes overlapping the locus in sense direction exhibited differential expression in “the same direction”, i.e. with same sign of log fold change, according to a binomial test (p < 0.05). Sources for annotations were equal to those used for probe design except for Gencode, where v14 used. Gencode was used as primary annotation and all non-overlapping annotations from the other sources were used in addition. Probes were considered, if at least 95% of its sequence overlapped with a particular annotation. Annotations were considered per gene, i.e. we considered overlaps with all exons of a gene and did not test for individual transcripts. If a probe mapped to multiple distinct annotated genes, we tested each gene individually but recorded the ambiguity to avoid losing potentially relevant signals. We considered only sense and discarded antisense overlap because the transcript structure is not known for transcripts that are antisense to annotated transcripts unless they map to known antisense transcripts, which were already included in the various annotation sources as listed above. However, for annotation items with an unknown reading direction (e.g., loci from the tiling array experiments, ncRNA predictions, caRNAs, we ignored the strand information and considered all overlaps.

For each probe p_i_ ϵ *s_diff_*, we determined whether p was located in a locus with known transcripts (protein-coding, non-coding, or pseudogenes, as described before). A probe was mapped to a particular gene if it was located in (i) an exon of at least one annotated splice variant (only for protein-coding transcripts because non-coding and pseudogene transcripts may exist in an unspliced and/or spliced version), (ii) the UTR of that gene, or (iii) in a putative previously unrecognized exon (no overlap with annotated exons but located in an exon of at least two spliced ESTs). Probes located exclusively intronic of a protein-coding gene (i.e., no overlap with annotated exons and less than two overlaps with exonic ESTs) were classified as putative intronic transcripts and therefore added to the non-coding list. If multiple introns overlapped, we used the cluster of overlapping introns as loci.

For each differentially expressed probe *pi ϵ s_diff_* that overlapped with a particular differential expression candidate i (i.e., a locus with known or unknown transcript structure) in sense direction, we then identified the set of probes *p*_alli_ that also overlapped with i with the criteria as described above (with respect to their genomic location such as exonic or intronic) and recorded the fraction of probes for which the expression level change was in the same direction as pi (i.e., up- or downregulated as compared to the control group). We then used a onetailed binomial test to identify differentially expressed loci with a significance threshold of p < 0.05. As this threshold can only be met with a minimal sample size of five probes, we separately recorded cases with less than five overlapping probes but more than 50% of the overlapping probes had a expression level change in the same direction.

Additionally, we recorded transcripts that achieved borderline significance (4 out of 5, 5 out of 6, and 6 out of 7 probes changing in the same direction). These loci should be treated with caution, however, because they may contain an increased amount of false positives. For probes located in loci with unknown transcript structures, we checked if the probe overlapped with spliced ESTs. If more than one spliced EST overlapped with the probe, we used the full overlapping EST cluster as locus rather than the original locus for the subsequent significance test. Lastly, for each of the four classes (three types of known transcripts and unknown transcripts), we filtered the list and only retained loci for which either the binomial test was significant or for which at least one probe had a differential expression q < 0.05. Although this procedure eliminates potentially relevant signals, it reduces the number of false positives due to the relatively high initial q-value.

**1.7. Computational Analyses of Splice Site Conservation**

Genome wide multiple sequence alignments across 18 vertebrate genomes (Supplementary tables S6 and S7) were used to construct a map of conserved splice sites as described in.^26^ Therefore we compiled a list of splice sites for each of the species defined by their annotated exon/intron boundaries. Based on human as reference, we evaluated the conservation of each splice sites in all of the other species present in the alignment. As in previous work ^26^ MaxEntScan scores ^27^ were used additionally to existing annotations to evaluate the conservation of splice sites. The splice site conservation rates of the consequently generated background sets could then be compared to the conservation rates of the signal sets, which were obtained as intersection of the background with the differentially expressed regions (Supplementary figure S1). An additional control set was obtained as intersection with regions that are expressed in human brain. We used brain expression data from the study of Necsulea et al. ^28^ here.

We stratified the data by the “degree of conservation” c, which is the fraction of conserved splice junctions per gene. We asked – for a fixed value of c – whether loci that are differentially expressed in AD patients show signs of accelerated evolution compared to the set of genes contained in the Gencode v14 annotation of the human genome. Different thresholds of c highlight different aspects of conservation and evolutionary change: At c > 0% we assay only presence or absence of a gene, and thus its evolutionary origin. The other extreme, c = 100% focusses on the complete precise conservation of the gene structure. We also show an intermediate level of gene structure conservation (c > 60%) in this supplement (Supplementary figures S3-S5).

**2. Supplementary Results**

We followed a multi-step approach to identify AD associated changes in gene expression: Initially, a whole genome tiling array was used to identify expressed regions in pooled AD and control samples, respectively. An Alzheimer Custom Microarray was designed, which interrogated the intervals identified as expressed in the tiling array approach, additional intervals found differentially expressed in response to several pathways and cell cycle described in ^5^ and additional ncRNA annotations from literature and databases. Subsequently, this custom array was applied to a set of AD and control samples for identifying AD-associated coding and non-coding genes.

**2.1. Tiling arrays identify expressed regions in AD and control samples**

We used whole genome tiling arrays to identify non-annotated transcripts in in three pooled AD and three pooled control samples, respectively. Using the TileShuffle algorithm we identified 64 488 and 48 412 transcribed fragments (transfrags) in AD and control samples, respectively, expressed significantly higher than background (FDR < 0.05). Again using TileShuffle, we found 1 459 transfrags that were significantly expressed in at least AD or control samples and significantly differentially expressed between both conditions (FDR < 0.05)

**2.2. Differentially expressed loci in Alzheimer’s disease**

Applying a custom expression microarray specifically designed for this study (see Supplementary Methods) to 17 AD and 19 control samples, we identified 4 184 probes differentially expressed between AD and control (q < 0.2). Of these, 4 095 mapped uniquely to the genome. Using a multi-step approach, we identified a set of 764 differentially expressed genomic loci, 31 of which were associated with at least three distinct differentially expressed probes. Dependent on the genomic location of the differentially expressed probe(s), we then associated each genomic locus with one of the four following classes: protein coding, non-coding, pseudogenes, and uncharacterized. The first three classes corresponded to known transcripts, whereas the latter represented loci with uncharacterized transcript structure and strand (see methods). In summary, we identified 162 differentially expressed protein-coding genes (Supplementary table S4), 460 differentially expressed non-coding genes or non-coding loci (Supplementary table S5), 29 differentially expressed pseudogenes, and 113 differentially expressed loci with unknown/uncharacterized transcript structure and type that did not overlap with any known genes or transcripts. The intersection of the identified differentially expressed loci with the constructed splice site conservation map of the Gencode-derived background, resulted in a set of 4 162 splice sites falling in 154 multi-exonic protein-coding transcripts and a set of 1 297 splice sites falling in 141 multi-exonic nonprotein-coding transcripts. Those data sets were used to compute the splice site conservation.

**2.3. No brain related bias in data**

When we employ the control set of brain-expressed human transcripts as background, all found results remain valid, since the difference in conservation rates compared to Gencode v14 annotated genes at large and the subset known to be expression in brain is marginal for all degrees of conservation c for both protein-coding and non-coding genes, (Supplementary figure S2).

References

1 Otto C, Reiche K, Hackermuller J. Detection of differentially expressed segments in tiling array data. *Bioinformatics* 2012; **28**: 1471–1479.

2 affymetrix.com. http://www.affymetrix.com/analysis/downloads/lf/tiling/Hs35b_MR_v02-2_NCBIv36_v2.bpmap.zip.

3 Benjamini Y, Hochberg Y. Controlling the False Discovery Rate: A Practical and Powerful Approach to Multiple Testing: Journal of the Royal Statistical Society. Series B (Methodological). *00359246* 1995; **57**: 289–300.

4 hgdownload.cse.ucsc.edu. http://hgdownload.cse.ucsc.edu/admin/exe/linux.x86_64/liftOver.

5 Hackermuller J, Reiche K, Otto C, Hosler N, Blumert C, Brocke-Heidrich K *et al.* Cell cycle, oncogenic and tumor suppressor pathways regulate numerous long and macro non-protein-coding RNAs. *Genome biology* 2014; **15**: R48.

6 Bu D, Yu K, Sun S, Xie C, Skogerbo G, Miao R *et al.* NONCODE v3.0: integrative annotation of long noncoding RNAs. *Nucleic acids research* 2012; **40**: D210-5.

7 Amaral PP, Clark MB, Gascoigne DK, Dinger ME, Mattick JS. lncRNAdb: a reference database for long noncoding RNAs. *Nucleic acids research* 2011; **39**: D146-51.

8 Kin T, Yamada K, Terai G, Okida H, Yoshinari Y, Ono Y *et al.* fRNAdb: a platform for mining/annotating functional RNA candidates from non-coding RNA sequences. *Nucleic acids research* 2007; **35**: D145-8.

9 Pang KC, Stephen S, Dinger ME, Engstrom PG, Lenhard B, Mattick JS. RNAdb 2.0--an expanded database of mammalian non-coding RNAs. *Nucleic acids research* 2007; **35**: D178-82.

10 Yamasaki C, Murakami K, Fujii Y, Sato Y, Harada E, Takeda J-i *et al.* The H-Invitational Database (H-InvDB), a comprehensive annotation resource for human genes and transcripts. *Nucleic acids research* 2008; **36**: D793-9.

11 Harrow J, Frankish A, Gonzalez JM, Tapanari E, Diekhans M, Kokocinski F *et al.* GENCODE: the reference human genome annotation for The ENCODE Project. *Genome research* 2012; **22**: 1760–1774.

12 Pruitt KD, Tatusova T, Brown GR, Maglott DR. NCBI Reference Sequences (RefSeq): current status, new features and genome annotation policy. *Nucleic acids research* 2012; **40**: D130-5.

13 Khalil AM, Guttman M, Huarte M, Garber M, Raj A, Rivea Morales D *et al.* Many human large intergenic noncoding RNAs associate with chromatin-modifying complexes and affect gene expression. *Proc Natl Acad Sci U S A* 2009; **106**: 11667–11672.

14 Mondal T, Rasmussen M, Pandey GK, Isaksson A, Kanduri C. Characterization of the RNA content of chromatin. *Genome research* 2010; **20**: 899–907.

15 Bartschat S, Kehr S, Tafer H, Stadler PF, Hertel J. snoStrip: a snoRNA annotation pipeline. *Bioinformatics* 2014; **30**: 115–116.

16 Nakaya HI, Amaral PP, Louro R, Lopes A, Fachel AA, Moreira YB *et al.* Genome mapping and expression analyses of human intronic noncoding RNAs reveal tissue-specific patterns and enrichment in genes related to regulation of transcription. *Genome biology* 2007; **8**: R43.

17 Gruber AR, Findeiss S, Washietl S, Hofacker IL, Stadler PF. RNAz 2.0: Improved noncoding RNA detection. *Pacific Symposium on Biocomputing. Pacific Symposium on Biocomputing* 2010: 69–79.

18 Pedersen JS, Bejerano G, Siepel A, Rosenbloom K, Lindblad-Toh K, Lander ES *et al.* Identification and classification of conserved RNA secondary structures in the human genome. *PLoS computational biology* 2006; **2**: e33.

19 Guil S, Esteller M. Cis-acting noncoding RNAs: friends and foes. *Nature structural & molecular biology* 2012; **19**: 1068–1075.

20 Arnold C, Externbrink F, Hackermuller J, Reiche K. CEM-designer: design of custom expression microarrays in the post-ENCODE Era. *Journal of biotechnology* 2014; **189**: 154–156.

21 Smyth GK. Linear models and empirical bayes methods for assessing differential expression in microarray experiments. *Statistical Applications in Genetics and Molecular Biology* 2004; **3**: Article3.

22 Bolstad BM, Irizarry RA, Astrand M, Speed TP. A comparison of normalization methods for high density oligonucleotide array data based on variance and bias. *Bioinformatics* 2003; **19**: 185–193.

23 Storey JD. A Direct Approach to False Discovery Rates: Journal of the Royal Statistical Society. Series B (Statistical Methodology). *13697412* 2002; **64**: 479–498.

24 Strimmer K. A unified approach to false discovery rate estimation. *BMC bioinformatics* 2008; **9**: 303.

25 Strimmer K. fdrtool: a versatile R package for estimating local and tail area-based false discovery rates. *Bioinformatics* 2008; **24**: 1461–1462.

26 Nitsche A, Rose D, Fasold M, Reiche K, Stadler PF. Comparison of splice sites reveals that long noncoding RNAs are evolutionarily well conserved. *RNA (New York, N.Y.)* 2015; **21**: 801–812.

27 Yeo G, Burge CB. Maximum entropy modeling of short sequence motifs with applications to RNA splicing signals. *Journal of computational biology a journal of computational molecular cell biology* 2004; **11**: 377–394.

28 Necsulea A, Soumillon M, Warnefors M, Liechti A, Daish T, Zeller U *et al.* The evolution of lncRNA repertoires and expression patterns in tetrapods. *Nature* 2014; **505**: 635–640.
