# Supplementary material for: Alzheimer-related genes show accelerated evolution
Source: Mol Psychiatry. 2020 Mar 13;26(10):5790–6. doi: 10.1038/s41380-020-0680-1 (PMC8758480; doi:10.1038/s41380-020-0680-1)

**Supplementary figure S1: Method workflow**. We constructed background sets from the Gencode v14 annotation (for protein-coding and long non-coding genes separately) and generated their splice site conservation maps with genome-wide multiple sequence alignment. The splice site conservation map for the AD-related genes is obtained as the intersection of the differentially expressed custom array loci with the background set. Empirical p-values are computed from sets of random loci of matching size drawn from the Gencode-derived background to evaluate the statistical significance.


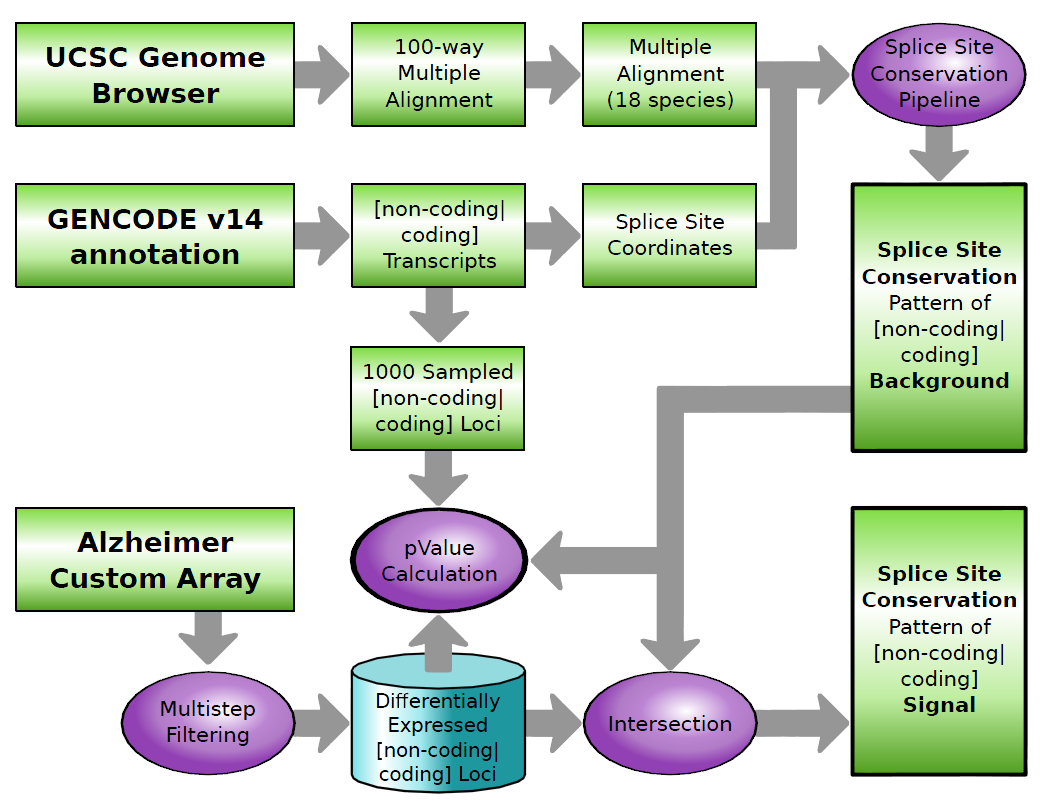


**Supplementary figure S2: Conservation rates of human brain-expressed non-coding and protein-coding genes** in comparison with the respective background of Gencode v14 annotated genes for different degrees of conservation (c > 0%, c = 100%).


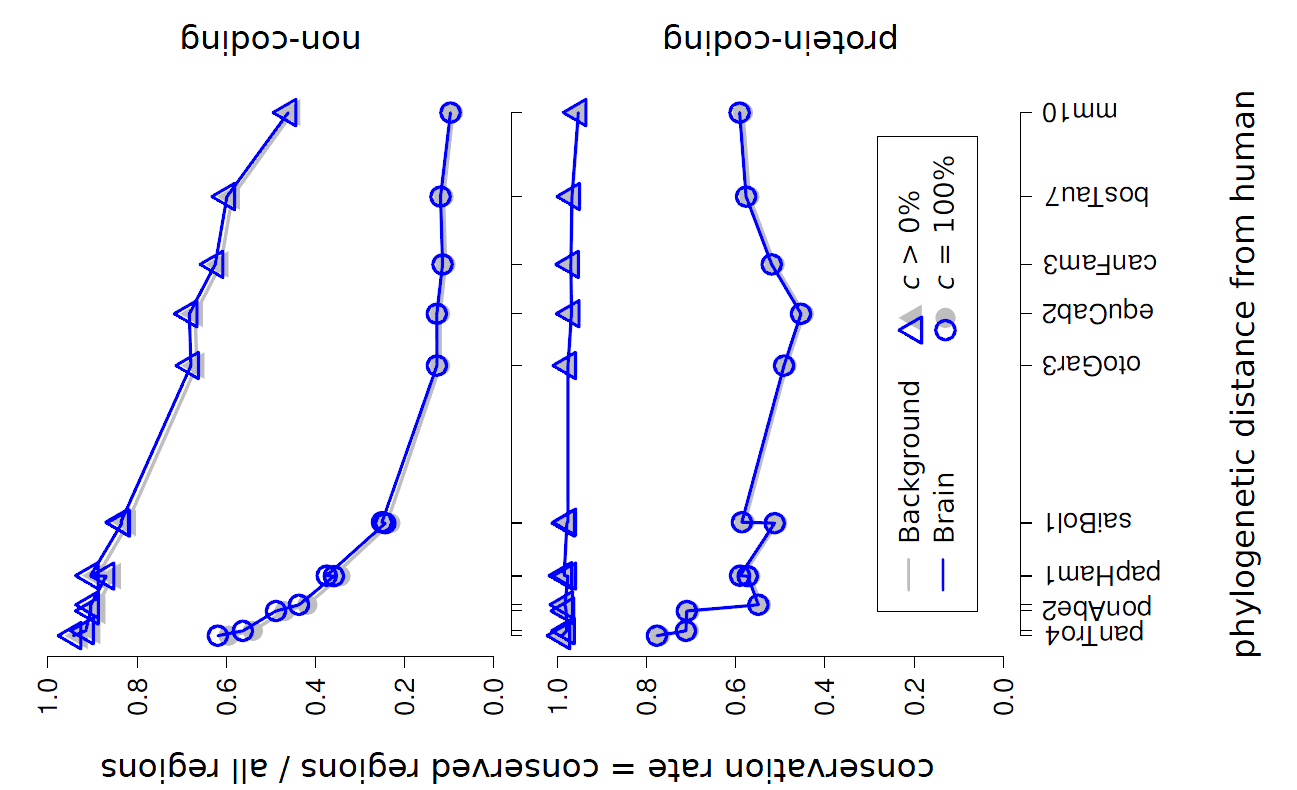


**Supplementary figure S3: Conservation rates of human AD-associated non-protein-coding (A-C) and protein-coding (D-F) regions** for different conservation degrees (c > 0%, c > 60%, c = 100%). On the horizontal axis mammalian species are indicated (denoted by the UCSC abbreviations) at their phylogenetic distance from human. Distinct data points are connected by lines to guide the eye. Variations in assembly and alignment quality cause some non-monotonicity in the curves, the overall decrease of conservation with phylogenetic distance is nevertheless clearly visible. Statistical significance of differences is computed independently for each species. Filled circles indicate p < 0.05. Even a moderate splice site turnover is much less common for protein-coding genes than for non-coding genes. This is reflected by the negligible differences between the conservation rates of signal and background for c > 60% (B, E).


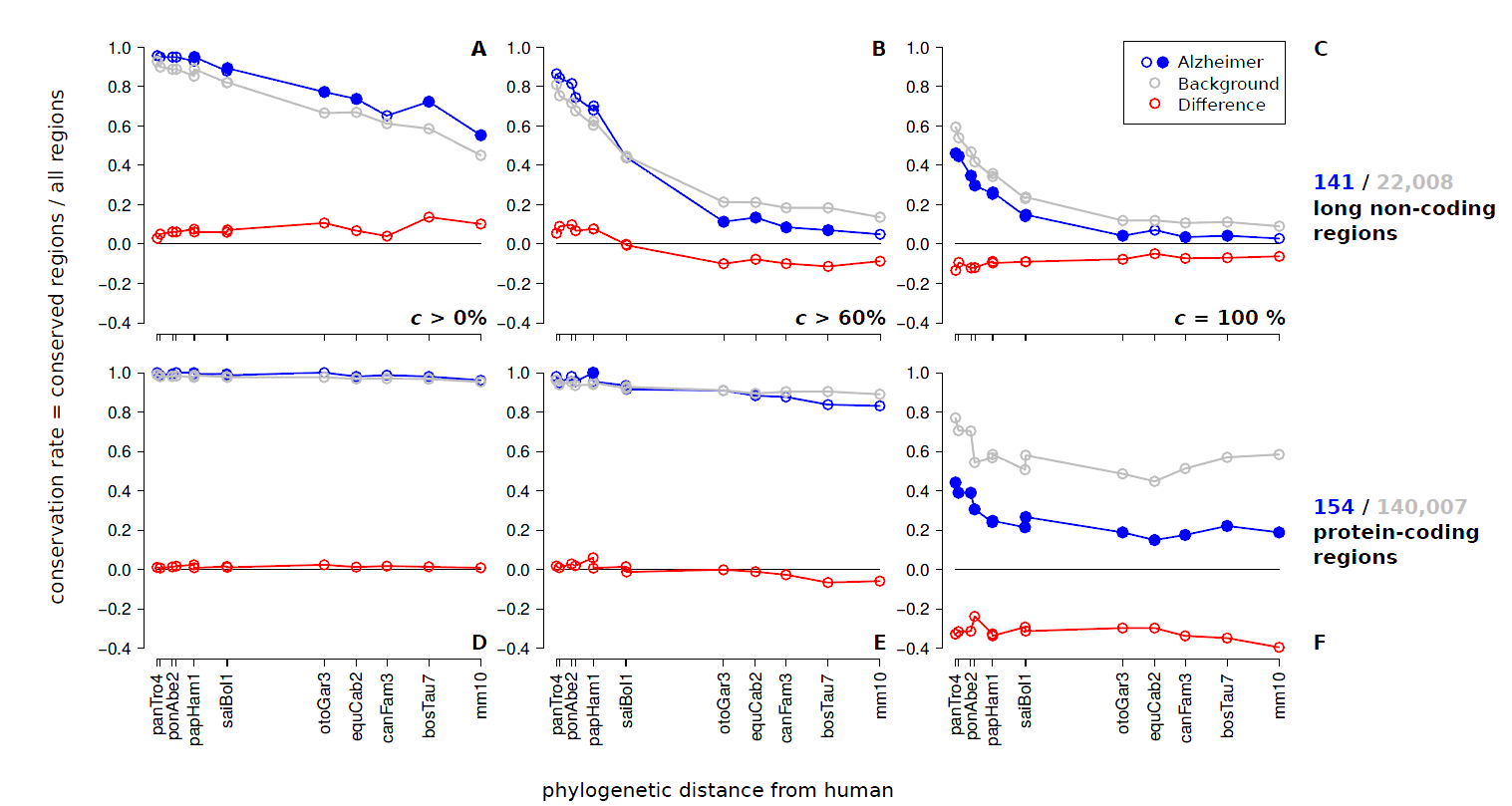


**Supplementary figure S4: Fraction of alignable human AD-associated non-protein-coding (A-C) and protein-coding (D-F) regions** for different degrees of alignability (a > 0%, a > 60%,a = 100%), that is the fraction of splice sites per region which are alignable to another species. The legend of this figure is analogous to Supplementary figure S3.


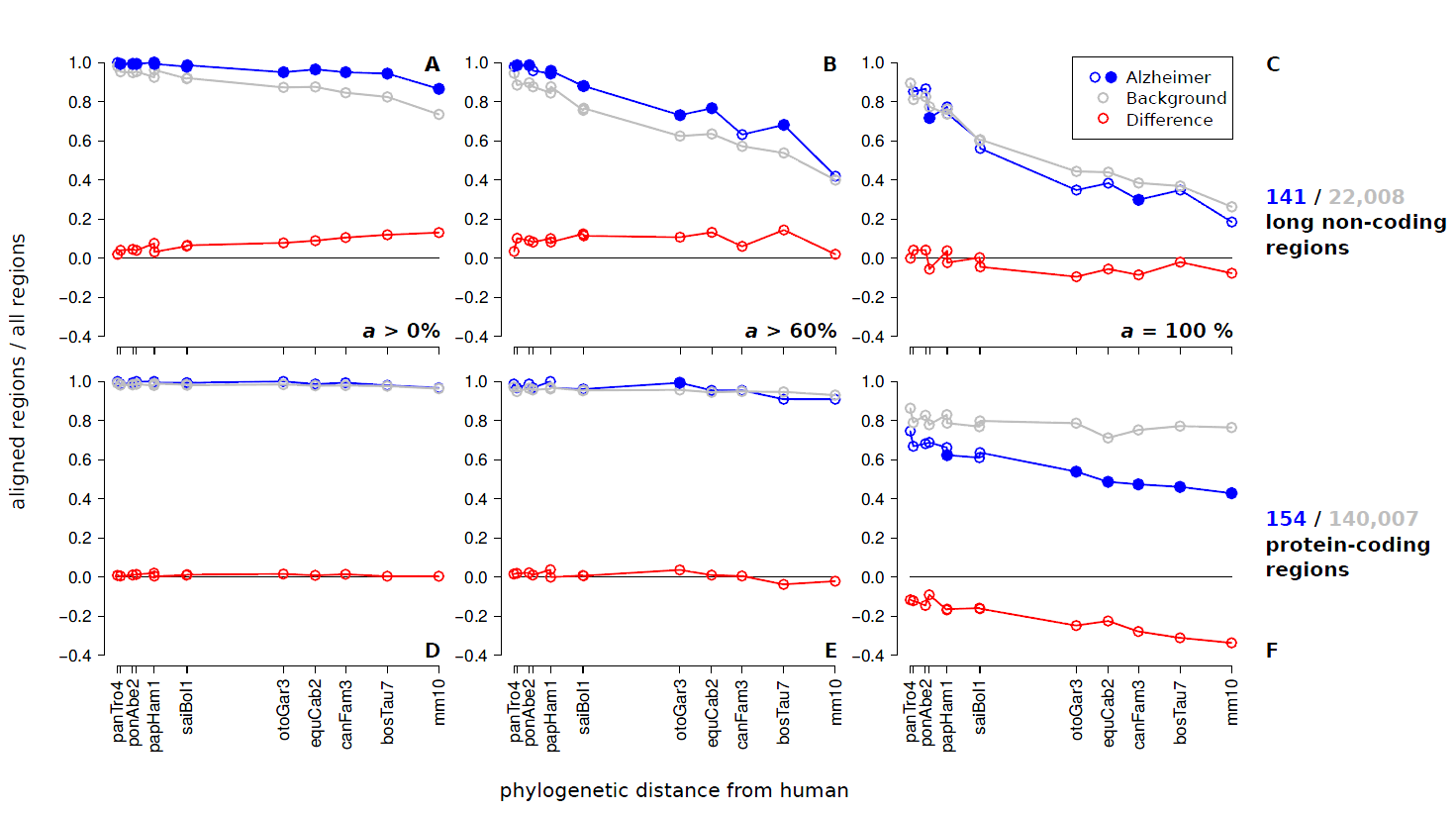


**Supplementary figure S5: Upper bound of conservation rates of human AD-associated non-protein-coding (A-C) and protein-coding (D-F) regions** for different conservation degrees (c > 0%, c > 60%, c = 100%). The amount of aligned regions is delimited by the degree of alignability a = c. The legend of this figure is analogous to Supplementary figure S3.


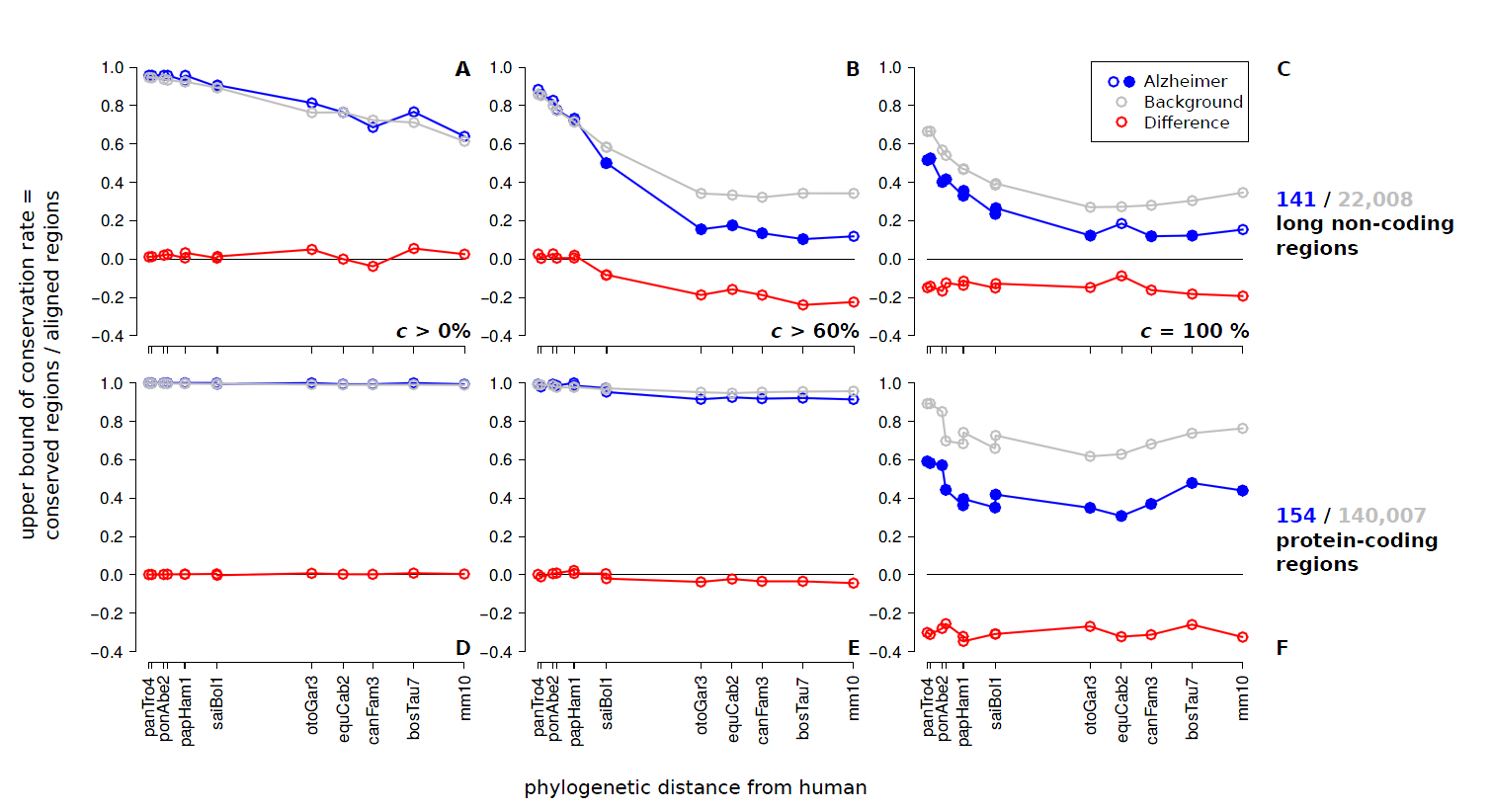

Supplement: Supplementary file 2 — Supplementary Figures S1-S5 [file 41380_2020_680_MOESM2_ESM.docx]
