# Supplementary material for: Alzheimer-related genes show accelerated evolution
Source: Mol Psychiatry. 2020 Mar 13;26(10):5790–6. doi: 10.1038/s41380-020-0680-1 (PMC8758480; doi:10.1038/s41380-020-0680-1)
Supplement: Supplementary file 3 — Supplementary Tables S1-S3 [file 41380_2020_680_MOESM3_ESM.docx]

**Supplementary table S1:** Genomic distribution of probes for the Alzheimer Custom Array, based on Gencode version 4, which was used for probe design. A probe corresponds to a category if it overlaps strand-specifically to at least 95% (57 nucleotides) with at least one annotation (i.e., feature or sequence) of the category. For introns and intergenic regions, the strand information has been ignored. 5’UTRs and 3’UTRs correspond to 5’ and 3’ untranslated regions of mRNAs. CDS corresponds to the coding exons of mRNAs. The relative fraction is defined according to overall number of probes on the Alzheimer Custom Array. The total numbers in the last column may not add up to 100% due to the mandatory control probes and probes that overlap with no category with at least 95%.

| **Annotation category** | **Number of probes** | **Relative fraction (in %)** |
| --- | --- | --- |
| 5’UTRs (sense) | 39,233 | 4.21 |
| 5’UTRs (antisense) | 38,021 | 4.08 |
| CDS (sense) | 70,451 | 7.56 |
| CDS (antisense) | 43,799 | 4.70 |
| 3’UTRs (sense) | 101,297 | 10.87 |
| 3’UTRs (antisense) | 73,340 | 7.87 |
| Introns | 388,881 | 41.73 |
| Intergenic regions | 162,803 | 17.47 |
| Pseudogenes | 8,201 | 0.88 |
| Repeats | 17,706 | 1.90 |

**Supplementary table S2:** According to the Gencode v14 lncRNA annotation the background and signal set of differentially expressed regions overlap with the following transcript annotations

| **Annotation** | **Number with Signal** | **Number in Background** |
| --- | --- | --- |
| 3prime_overlapping_ncrna | 23 | 0 |
| ambiguous_orf | 19 | 0 |
| antisense | 6954 | 209 |
| lincRNA | 8020 | 103 |
| non_coding | 6 | 1 |
| processed_transcript | 4086 | 134 |
| retained_intron | 410 | 8 |
| sense_intronic | 546 | 11 |
| sense_overlapping | 72 | 1 |
| TEC | 1 | 1 |

**Supplementary table S****3:** Overlap with known AD-associated genes and genes associated with other brain diseases. The table lists all differentially expressed loci that overlap either in sense or antisense direction with known AD-associated genes or genes associated with other brain diseases according to various references.

Gene: Gene name with reported AD or brain disease association as reported by the reference. Ensembl ID: Ensembl ID of the Gene. Signal origin: Characterization of the location from which the signal originates from in custom arrays. Overlap direction: Direction of overlap with the reported gene from the literature.

________________________________________________________________________________________

Gene Ensembl ID Signal origin Overlap associated Reference

direction brain

diseases

________________________________________________________________________________________

PLPPR4 ENSG00000117600 intron of protein-coding gene sense IDS ^1^

CALHM1 ENSG00000185933 exon of protein-coding gene sense AD ^2^

SEC23IP ENSG00000107651 exon of protein-coding gene sense NDD ^3^

WDR11 ENSG00000120008 exon of protein-coding gene sense AD ^4^

SLC1A2 ENSG00000110436 exon of protein-coding gene sense AD ^5^

SCN9A ENSG00000169432 exon of protein-coding gene sense PNP ^6^

KCNIP4 ENSG00000185774 intron of protein-coding gene sense AD ^7^

GRID2 ENSG00000152208 exon of protein-coding gene sense BA ^8^

CASK ENSG00000147044 exon of protein-coding gene sense NDD ^9^

PLCH2 ENSG00000149527 exon of protein-coding gene sense NDD ^10^

MMEL1 ENSG00000142606 exon of protein-coding gene sense AD ^11^

FOXD3 ENSG00000187140 exon of protein-coding gene sense MDD ^12^

SYPL2 ENSG00000143028 intron of protein-coding gene sense AD ^13^

FCRL5 ENSG00000143297 intron of protein-coding gene sense AD ^14^

CTSE ENSG00000196188 intron of protein-coding gene sense AD ^15^

HHAT ENSG00000054392 exon of protein-coding gene sense IDS ^16^

RYR2 ENSG00000198626 intron of protein-coding gene sense AD ^17^

MKI67 ENSG00000148773 exon of protein-coding gene sense AD ^18^

DDI1 ENSG00000170967 exon of protein-coding gene sense AD ^19^

CASP12 ENSG00000204403 exon of protein-coding gene sense AD ^20^

CCDC60 ENSG00000183273 exon of protein-coding gene sense SCZ ^21^

FREM2 ENSG00000150893 exon of protein-coding gene sense NTDD ^22^

DLEU1 ENSG00000176124 intron of protein-coding gene sense MS ^23^

FERMT2 ENSG00000073712 exon of protein-coding gene sense AD ^24^

CASC5 ENSG00000137812 exon of protein-coding gene sense PMENC ^25^

WDR76 ENSG00000092470 exon of protein-coding gene sense AD ^26^

ANXA2 ENSG00000182718 intron of protein-coding gene sense AD ^27^

ABI3 ENSG00000108798 exon of protein-coding gene sense AD ^28^

APOH ENSG00000091583 exon of protein-coding gene sense MCI, SCZ ^29^, ^30^

KIAA1328 ENSG00000150477 exon of protein-coding gene sense IDS ^31^

GPR17 ENSG00000144230 exon of protein-coding gene sense AD, MS ^32^, ^33^

COL3A1 ENSG00000168542 exon of protein-coding gene sense DS ^34^

SDC4 ENSG00000124145 exon of protein-coding gene sense AD ^35^

APOL4 ENSG00000100336 exon of protein-coding gene sense SCZ ^36^

NPTXR ENSG00000221890 exon of protein-coding gene sense AD ^37^, ^38^

CELSR1 ENSG00000075275 intron of protein-coding gene sense STR, SB ^39^, ^40^

RARB ENSG00000077092 intron of protein-coding gene sense SCZ ^41^

EOMES ENSG00000163508 exon of protein-coding gene sense AD ^42^

ITIH1 ENSG00000055957 intron of protein-coding gene sense SCZ ^43^

FHIT ENSG00000189283 exon of protein-coding gene sense DEP ^44^

ZIC4 ENSG00000174963 exon of protein-coding gene sense AD ^45^

ZIC1 ENSG00000152977 exon of protein-coding gene sense MEC, LD ^46^,^47^

DSPP ENSG00000152591 exon of protein-coding gene sense CD ^48^

AGXT2L1 ENSG00000164089 exon of protein-coding gene sense SCZ, BD ^49^

SH3RF1 ENSG00000154447 exon of protein-coding gene sense TLE ^50^

PCDH12 ENSG00000113555 exon of protein-coding gene sense EPI ^51^

FAT2 ENSG00000086570 exon of protein-coding gene sense SA ^52^

TFAP2A ENSG00000137203 exon of protein-coding gene sense AD ^53^

BTBD9 ENSG00000183826 exon of protein-coding gene sense RLS ^54^

CD36 ENSG00000135218 exon of protein-coding gene sense AD ^55^

TGFBR1 ENSG00000106799 exon of protein-coding gene sense AD ^56^

TOR4A ENSG00000198113 exon of protein-coding gene sense NPC ^57^

MXRA5 ENSG00000101825 exon of protein-coding gene sense ASD ^58^

GLRA2 ENSG00000101958 exon of protein-coding gene sense ASD ^59^

APOA2 ENSG00000158874 exon of protein-coding gene sense AD, MC I ^60^, ^29^

MME ENSG00000196549 intron of protein-coding gene sense AD, APN ^60^, ^61^

MYOZ3 ENSG00000164591 exon of protein-coding gene sense AD ^62^

GRIA1 ENSG00000155511 intron of protein-coding gene sense AD, SCZ ^62^, ^63^

NEDD9 ENSG00000111859 intron of protein-coding gene sense AD ^64^

ADCYAP1R1 ENSG00000078549 intron of protein-coding gene sense AD ^60^

CAV1 ENSG00000105974 intron of protein-coding gene sense AD, ASD ^60^, ^65^

NRG1 ENSG00000157168 intron of protein-coding gene sense AD, SCZ ^60^, ^66^

SLC18A3 ENSG00000187714 exon of protein-coding gene sense AD, PSP ^60^, ^67^

CALHM1 ENSG00000185933 exon of protein-coding gene sense AD ^60^

TCF7L2 ENSG00000148737 intron of protein-coding gene sense AD, BD ^60^, ^68^

ADAM12 ENSG00000148848 intron of protein-coding gene sense AD, SCZ ^60^, ^69^

BDNF ENSG00000176697 exon of protein-coding gene sense AD, PD, HD ^60^, ^70^, ^71^

ATXN8OS ENSG00000230223 antisense lincRNA sense AD, ALS ^60^, ^72^

SAMD4A ENSG00000020577 intron of protein-coding gene sense AD ^73^

FOS ENSG00000170345 intron of protein-coding gene sense AD ^64^

TGFB3 ENSG00000119699 exon of protein-coding gene sense AD, MS ^73^, ^74^

LIPC ENSG00000166035 intron of protein-coding gene sense AD ^60^

IGF1R ENSG00000140443 intron of protein-coding gene sense AD, HAND ^60^, ^75^

TP53 ENSG00000141510 exon +intron of protein-coding sense AD ^60^

gene

RUNX1 ENSG00000159216 intron of protein-coding gene sense AD ^60^

RBM3 ENSG00000102317 exon of protein-coding gene sense AD ^73^

STARD7 ENSG00000084090 intron of protein-coding gene antisense AD ^62^

TTN ENSG00000237298 antisense lincRNA antisense AD ^76^

EFNA5 ENSG00000184349 RNAz prediction, intronic ncRNA antisense AD, PD, SCZ ^60^, ^77^

ABCB1 ENSG00000085563 exon/intron boundary of protein- antisense AD, DEP ^60^, ^78^

coding gene

SLC22A18 ENSG00000254827 intron of protein-coding gene antisense AD ^62^

GAB2 ENSG00000254420 antisense lincRNA antisense AD ^60^

C18orf10 ENSG00000150477 exon of protein-coding gene antisense AD ^62^

MCM3AP ENSG00000215424 antisense lincRNA antisense AD, CMTN ^60^, ^79^

AD, Alzheimer’s disease; ALS, Amyotrophic lateral sclerosis; APN, Axonal polyneuropathy; ASD, Autism spectrum disorder; BA, Brain atrophy; BD, Bipolar disorder; CD, Cognitive deficits; CMTN, Charcot-Marie-Tooth neuropathy; DEP, Depression; DS, Down syndrome; HAND, HIV-associated neurocognitive disorders; HD, Huntington’s disease; IDS, Intellectual disability; LD, Learning disability; MCI, Mild cognitive impairment; MDD, Major depressive disorder; MEC, Microcephaly; MS, Multiple sclerosis; NDD, Neurodevelopmental disorder; NPC, Niemann-Pick disease type C; NTDD, Neural tube defect disorders; PD, Parkinson’s disease; PMENC, Primary microcephaly; PNP, Peripheral neuropathy; PSP, Progressive supranuclear palsy; RLS, Restless leg syndrome; SA, Spinocerebellar ataxia; SB, Spina bifida; SCZ, Schizophrenia; STR, Stroke; TLE, Intractable temporal epilepsy;

References

1 Chen X, Li H, Chen C, Zhou L, Xu X, Xiang Y *et al.* Genome-Wide Array Analysis Reveals Novel Genomic Regions and Candidate Gene for Intellectual Disability. *Molecular diagnosis & therapy* 2018; **22**: 749–757.

2 Mun M-J, Kim J-H, Choi J-Y, Jang W-C. Calcium homeostasis modulator 1 gene P86L polymorphism and the risk for alzheimer's disease: A meta-analysis. *Neuroscience letters* 2016; **619**: 8–14.

3 Reuter MS, Tawamie H, Buchert R, Hosny Gebril O, Froukh T, Thiel C *et al.* Diagnostic Yield and Novel Candidate Genes by Exome Sequencing in 152 Consanguineous Families With Neurodevelopmental Disorders. *JAMA psychiatry* 2017; **74**: 293–299.

4 Hohman TJ, Koran MEI, Thornton-Wells TA. Genetic variation modifies risk for neurodegeneration based on biomarker status. *Frontiers in Aging Neuroscience* 2014; **6**: 183.

5 Masliah E, Alford M, Deteresa R, Mallory M, Hansen L. Deficient glutamate transport is associated with neurodegeneration in Alzheimer's disease. *Annals of neurology* 1996; **40**: 759–766.

6 Sibon I, Toffol B de, Azulay J-P, Sellal F, Thomas-Antérion C, Léger J-M *et al.* American Academy of Neurology, Washington, 18-25 avril 2015. *Revue neurologique* 2015; **171**: 581–601.

7 Massone S, Vassallo I, Castelnuovo M, Fiorino G, Gatta E, Robello M *et al.* RNA polymerase III drives alternative splicing of the potassium channel-interacting protein contributing to brain complexity and neurodegeneration. *J Cell Biol* 2011; **193**: 851–866.

8 Ali Z, Zulfiqar S, Klar J, Wikström J, Ullah F, Khan A *et al.* Homozygous GRID2 missense mutation predicts a shift in the D-serine binding domain of GluD2 in a case with generalized brain atrophy and unusual clinical features. *BMC medical genetics* 2017; **18**: 144.

9 Popp B, Ekici AB, Thiel CT, Hoyer J, Wiesener A, Kraus C *et al.* Exome Pool-Seq in neurodevelopmental disorders. *European journal of human genetics EJHG* 2017; **25**: 1364–1376.

10 Lo Vasco VR. Role of phosphoinositide-specific phospholipase C η2 in isolated and syndromic mental retardation. *European neurology* 2011; **65**: 264–269.

11 Huang JY, Hafez DM, James BD, Bennett DA, Marr RA. Altered NEP2 expression and activity in mild cognitive impairment and Alzheimer's disease. *Journal of Alzheimer's disease JAD* 2012; **28**: 433–441.

12 Kang HJ, Adams DH, Simen A, Simen BB, Rajkowska G, Stockmeier CA *et al.* Gene expression profiling in postmortem prefrontal cortex of major depressive disorder. *The Journal of neuroscience the official journal of the Society for Neuroscience* 2007; **27**: 13329–13340.

13 Chen S, Lu FF, Seeman P, Liu F. Quantitative proteomic analysis of human substantia nigra in Alzheimer's disease, Huntington's disease and Multiple sclerosis. *Neurochemical research* 2012; **37**: 2805–2813.

14 Velez JI, Lopera F, Sepulveda-Falla D, Patel HR, Johar AS, Chuah A *et al.* APOE*E2 allele delays age of onset in PSEN1 E280A Alzheimer's disease. *Molecular psychiatry* 2016; **21**: 916–924.

15 Bernstein HG, Wiederanders B. An immunohistochemical study of cathepsin E in Alzheimer-type dementia brains. *Brain research* 1994; **667**: 287–290.

16 Agha Z, Iqbal Z, Azam M, Ayub H, Vissers LELM, Gilissen C *et al.* Exome sequencing identifies three novel candidate genes implicated in intellectual disability. *PLoS One* 2014; **9**: e112687.

17 Del Prete D, Checler F, Chami M. Ryanodine receptors: physiological function and deregulation in Alzheimer disease. *Mol Neurodegener* 2014; **9**: 21.

18 Smith TW, Lippa CF. Ki-67 immunoreactivity in Alzheimer's disease and other neurodegenerative disorders. *J Neuropathol Exp Neurol* 1995; **54**: 297–303.

19 Alexander J, Kalev O, Mehrabian S, Traykov L, Raycheva M, Kanakis D *et al.* Familial early-onset dementia with complex neuropathologic phenotype and genomic background. *Neurobiology of aging* 2016; **42**: 199–204.

20 Mehmet H. Caspases find a new place to hide. *Nature* 2000; **403**: 29–30.

21 Kirov G, Zaharieva I, Georgieva L, Moskvina V, Nikolov I, Cichon S *et al.* A genome-wide association study in 574 schizophrenia trios using DNA pooling. *Molecular psychiatry* 2009; **14**: 796–803.

22 Beaumont M, Akloul L, Carré W, Quélin C, Journel H, Pasquier L *et al.* Targeted panel sequencing establishes the implication of planar cell polarity pathway and involves new candidate genes in neural tube defect disorders. *Human genetics* 2019; **138**: 363–374.

23 Andlauer TFM, Buck D, Antony G, Bayas A, Bechmann L, Berthele A *et al.* Novel multiple sclerosis susceptibility loci implicated in epigenetic regulation. *Science advances* 2016; **2**: e1501678.

24 Boscher E, Husson T, Quenez O, Laquerrière A, Marguet F, Cassinari K *et al.* Copy Number Variants in miR-138 as a Potential Risk Factor for Early-Onset Alzheimer's Disease. *Journal of Alzheimer's disease JAD* 2019; **68**: 1243–1255.

25 Genin A, Desir J, Lambert N, Biervliet M, van der Aa N, Pierquin G *et al.* Kinetochore KMN network gene CASC5 mutated in primary microcephaly. *Human molecular genetics* 2012; **21**: 5306–5317.

26 Raghavan NS, Brickman AM, Andrews H, Manly JJ, Schupf N, Lantigua R *et al.* Whole-exome sequencing in 20,197 persons for rare variants in Alzheimer's disease. *Annals of clinical and translational neurology* 2018; **5**: 832–842.

27 Eberhard DA, Brown MD, VandenBerg SR. Alterations of annexin expression in pathological neuronal and glial reactions. Immunohistochemical localization of annexins I, II (p36 and p11 subunits), IV, and VI in the human hippocampus. *The American journal of pathology* 1994; **145**: 640–649.

28 Sims R, van der Lee SJ, Naj AC, Bellenguez C, Badarinarayan N, Jakobsdottir J *et al.* Rare coding variants in PLCG2, ABI3, and TREM2 implicate microglial-mediated innate immunity in Alzheimer's disease. *Nature genetics* 2017; **49**: 1373–1384.

29 Song F, Poljak A, Crawford J, Kochan NA, Wen W, Cameron B *et al.* Plasma apolipoprotein levels are associated with cognitive status and decline in a community cohort of older individuals. *PLoS One* 2012; **7**: e34078.

30 Balan S, Iwayama Y, Toyota T, Toyoshima M, Maekawa M, Yoshikawa T. 22q11.2 deletion carriers and schizophrenia-associated novel variants. *The British journal of psychiatry the journal of mental science* 2014; **204**: 398–399.

31 Barone R, Fichera M, Grandi M de, Battaglia M, Lo Faro V, Mattina T *et al.* Familial 18q12.2 deletion supports the role of RNA-binding protein CELF4 in autism spectrum disorders. *American journal of medical genetics. Part A* 2017; **173**: 1649–1655.

32 Saravanan KM, Palanivel S, Yli-Harja O, Kandhavelu M. Identification of novel GPR17-agonists by structural bioinformatics and signaling activation. *International journal of biological macromolecules* 2018; **106**: 901–907.

33 Alavi MS, Karimi G, Roohbakhsh A. The role of orphan G protein-coupled receptors in the pathophysiology of multiple sclerosis: A review. *Life sciences* 2019; **224**: 33–40.

34 Chung I-H, Lee S-H, Lee K-W, Park S-h, Cha K-Y, Kim N-S *et al.* Gene expression analysis of cultured amniotic fluid cell with Down syndrome by DNA microarray. *Journal of Korean medical science* 2005; **20**: 82–87.

35 Salza R, Lethias C, Ricard-Blum S. The Multimerization State of the Amyloid-β42 Amyloid Peptide Governs its Interaction Network with the Extracellular Matrix. *Journal of Alzheimer's disease JAD* 2017; **56**: 991–1005.

36 Takahashi S, Cui Y-h, Han Y-h, Fagerness JA, Galloway B, Shen Y-c *et al.* Association of SNPs and haplotypes in APOL1, 2 and 4 with schizophrenia. *Schizophr Res* 2008; **104**: 153–164.

37 Begcevic I, Tsolaki M, Brinc D, Brown M, Martinez-Morillo E, Lazarou I *et al.* Neuronal pentraxin receptor-1 is a new cerebrospinal fluid biomarker of Alzheimer's disease progression. *F1000Research* 2018; **7**: 1012.

38 Hendrickson RC, Lee AYH, Song Q, Liaw A, Wiener M, Paweletz CP *et al.* High Resolution Discovery Proteomics Reveals Candidate Disease Progression Markers of Alzheimer's Disease in Human Cerebrospinal Fluid. *PLoS One* 2015; **10**: e0135365.

39 Lei Y, Zhu H, Yang W, Ross ME, Shaw GM, Finnell RH. Identification of novel CELSR1 mutations in spina bifida. *PLoS One* 2014; **9**: e92207.

40 Zhan Y-H, Lin Y, Tong S-J, Ma Q-L, Lu C-X, Fang L *et al.* The CELSR1 polymorphisms rs6007897 and rs4044210 are associated with ischaemic stroke in Chinese Han population. *Annals of human biology* 2015; **42**: 26–30.

41 Reay WR, Atkins JR, Quidé Y, Carr VJ, Green MJ, Cairns MJ. Polygenic disruption of retinoid signalling in schizophrenia and a severe cognitive deficit subtype. *Molecular psychiatry* 2018.

42 Khondoker M, Newhouse S, Westman E, Muehlboeck J-S, Mecocci P, Vellas B *et al.* Linking Genetics of Brain Changes to Alzheimer's Disease: Sparse Whole Genome Association Scan of Regional MRI Volumes in the ADNI and AddNeuroMed Cohorts. *Journal of Alzheimer's disease JAD* 2015; **45**: 851–864.

43 Witt SH, Juraeva D, Sticht C, Strohmaier J, Meier S, Treutlein J *et al.* Investigation of manic and euthymic episodes identifies state- and trait-specific gene expression and STAB1 as a new candidate gene for bipolar disorder. *Translational psychiatry* 2014; **4**: e426.

44 Direk N, Williams S, Smith JA, Ripke S, Air T, Amare AT *et al.* An Analysis of Two Genome-wide Association Meta-analyses Identifies a New Locus for Broad Depression Phenotype. *Biological psychiatry* 2017; **82**: 322–329.

45 Zhao J, Cheng F, Jia P, Cox N, Denny JC, Zhao Z. An integrative functional genomics framework for effective identification of novel regulatory variants in genome-phenome studies. *Genome medicine* 2018; **10**: 7.

46 Vandervore LV, Schot R, Hoogeboom AJM, Lincke C, Coo IF de, Lequin MH *et al.* Mutated zinc finger protein of the cerebellum 1 leads to microcephaly, cortical malformation, callosal agenesis, cerebellar dysplasia, tethered cord and scoliosis. *European journal of medical genetics* 2018; **61**: 783–789.

47 Twigg SRF, Forecki J, Goos JAC, Richardson ICA, Hoogeboom AJM, van den Ouweland AMW *et al.* Gain-of-Function Mutations in ZIC1 Are Associated with Coronal Craniosynostosis and Learning Disability. *American journal of human genetics* 2015; **97**: 378–388.

48 Iourov IY, Zelenova MA, Vorsanova SG, Voinova VV, Yurov YB. 4q21.2q21.3 Duplication: Molecular and Neuropsychological Aspects. *Current genomics* 2018; **19**: 173–178.

49 Shao L, Vawter MP. Shared gene expression alterations in schizophrenia and bipolar disorder. *Biological psychiatry* 2008; **64**: 89–97.

50 Wang X, Tian X, Yang Y, Lu X, Li Y, Ma Y *et al.* POSH participates in epileptogenesis by increasing the surface expression of the NMDA receptor: a promising therapeutic target for epilepsy. *Expert opinion on therapeutic targets* 2017; **21**: 1083–1094.

51 Suzuki-Muromoto S, Wakusawa K, Miyabayashi T, Sato R, Okubo Y, Endo W *et al.* A case of new PCDH12 gene variants presented as dyskinetic cerebral palsy with epilepsy. *Journal of human genetics* 2018; **63**: 749–753.

52 Nibbeling EAR, Duarri A, Verschuuren-Bemelmans CC, Fokkens MR, Karjalainen JM, Smeets CJLM *et al.* Exome sequencing and network analysis identifies shared mechanisms underlying spinocerebellar ataxia. *Brain a journal of neurology* 2017; **140**: 2860–2878.

53 Lahiri DK. Apolipoprotein E as a target for developing new therapeutics for Alzheimer's disease based on studies from protein, RNA, and regulatory region of the gene. *J.Mol.Neurosci.* 2004; **23**: 225–233.

54 Assimakopoulos K, Karaivazoglou K, Skokou M, Kalogeropoulou M, Kolios P, Gourzis P *et al.* Genetic Variations Associated with Sleep Disorders in Patients with Schizophrenia: A Systematic Review. *Medicines (Basel, Switzerland)* 2018; **5**.

55 Šerý O, Janoutová J, Ewerlingová L, Hálová A, Lochman J, Janout V *et al.* CD36 gene polymorphism is associated with Alzheimer's disease. *Biochimie* 2017; **135**: 46–53.

56 Lippa CF, Flanders KC, Kim ES, Croul S. TGF-beta receptors-I and -II immunoexpression in Alzheimer's disease: a comparison with aging and progressive supranuclear palsy. *Neurobiol.Aging* 1998; **19**: 527–533.

57 Rauniyar N, Subramanian K, Lavallée-Adam M, Martínez-Bartolomé S, Balch WE, Yates JR. Quantitative Proteomics of Human Fibroblasts with I1061T Mutation in Niemann-Pick C1 (NPC1) Protein Provides Insights into the Disease Pathogenesis. *Molecular & cellular proteomics MCP* 2015; **14**: 1734–1749.

58 Al-Mubarak B, Abouelhoda M, Omar A, AlDhalaan H, Aldosari M, Nester M *et al.* Whole exome sequencing reveals inherited and de novo variants in autism spectrum disorder: a trio study from Saudi families. *Scientific reports* 2017; **7**: 5679.

59 Lin M-S, Xiong W-C, Li S-J, Gong Z, Cao X, Kuang X-J *et al.* α2-glycine receptors modulate adult hippocampal neurogenesis and spatial memory. *Developmental neurobiology* 2017; **77**: 1430–1441.

60 Bertram L, McQueen MB, Mullin K, Blacker D, Tanzi RE. Systematic meta-analyses of Alzheimer disease genetic association studies: the AlzGene database. *Nature genetics* 2007; **39**: 17–23.

61 Auer-Grumbach M, Toegel S, Schabhuttl M, Weinmann D, Chiari C, Bennett DLH *et al.* Rare Variants in MME, Encoding Metalloprotease Neprilysin, Are Linked to Late-Onset Autosomal-Dominant Axonal Polyneuropathies. *American journal of human genetics* 2016; **99**: 607–623.

62 Bossers K, Wirz KT, Meerhoff GF, Essing AH, van Dongen JW, Houba P *et al.* Concerted changes in transcripts in the prefrontal cortex precede neuropathology in Alzheimer's disease. *Brain.* 2010; **133**: 3699–3723.

63 Sundararajan T, Manzardo AM, Butler MG. Functional analysis of schizophrenia genes using GeneAnalytics program and integrated databases. *Gene* 2018; **641**: 25–34.

64 Ginsberg SD, Alldred MJ, Counts SE, Cataldo AM, Neve RL, Jiang Y *et al.* Microarray analysis of hippocampal CA1 neurons implicates early endosomal dysfunction during Alzheimer's disease progression. *Biological psychiatry* 2010; **68**: 885–893.

65 Limpitikul WB, Dick IE, Ben-Johny M, Yue DT. An autism-associated mutation in CaV1.3 channels has opposing effects on voltage- and Ca(2+)-dependent regulation. *Scientific reports* 2016; **6**: 27235.

66 Nawaz R, Asif H, Khan A, Ishtiaq H, Shad F, Siddiqui S. Drugs targeting SNPrs35753505 of the NRG1 gene may prevent the association of neurological disorder schizophrenia in a Pakistani population. *CNS.Neurol.Disord.Drug Targets.* 2014; **13**: 1604–1614.

67 Suzuki M, Desmond TJ, Albin RL, Frey KA. Cholinergic vesicular transporters in progressive supranuclear palsy. *Neurology* 2002; **58**: 1013–1018.

68 Cuellar-Barboza AB, Winham SJ, McElroy SL, Geske JR, Jenkins GD, Colby CL *et al.* Accumulating evidence for a role of TCF7L2 variants in bipolar disorder with elevated body mass index. *Bipolar disorders* 2016; **18**: 124–135.

69 Farkas N, Lendeckel U, Dobrowolny H, Funke S, Steiner J, Keilhoff G *et al.* Reduced density of ADAM 12-immunoreactive oligodendrocytes in the anterior cingulate white matter of patients with schizophrenia. *The world journal of biological psychiatry the official journal of the World Federation of Societies of Biological Psychiatry* 2010; **11**: 556–566.

70 Colangelo V, Schurr J, Ball MJ, Pelaez RP, Bazan NG, Lukiw WJ. Gene expression profiling of 12633 genes in Alzheimer hippocampal CA1: transcription and neurotrophic factor down-regulation and up-regulation of apoptotic and pro-inflammatory signaling. *J.Neurosci.Res.* 2002; **70**: 462–473.

71 Numakawa T, Odaka H, Adachi N. Actions of Brain-Derived Neurotrophin Factor in the Neurogenesis and Neuronal Function, and Its Involvement in the Pathophysiology of Brain Diseases. *International journal of molecular sciences* 2018; **19**.

72 Hirano M, Samukawa M, Isono C, Saigoh K, Nakamura Y, Kusunoki S. Noncoding repeat expansions for ALS in Japan are associated with the ATXN8OS gene. *Neurology. Genetics* 2018; **4**: e252.

73 Tan MG, Chua W-T, Esiri MM, Smith AD, Vinters HV, Lai MK. Genome wide profiling of altered gene expression in the neocortex of Alzheimer's disease. *J Neurosci Res* 2010; **88**: 1157–1169.

74 Hassanzadeh G, Hosseini Quchani S, Sahraian MA, Abolhassani F, Sadighi Gilani MA, Dehghan Tarzjani M *et al.* Leukocyte Gene Expression and Plasma Concentration in Multiple Sclerosis: Alteration of Transforming Growth Factor-betas, Claudin-11, and Matrix Metalloproteinase-2. *Cellular and molecular neurobiology* 2016; **36**: 865–872.

75 Gerena Y, Menendez-Delmestre R, Skolasky RL, Hechavarria RM, Perez S, Hilera C *et al.* Soluble insulin receptor as a source of insulin resistance and cognitive impairment in HIV-seropositive women. *Journal of neurovirology* 2015; **21**: 113–119.

76 Gomez Ravetti M, Rosso OA, Berretta R, Moscato P. Uncovering molecular biomarkers that correlate cognitive decline with the changes of hippocampus' gene expression profiles in Alzheimer's disease. *PloS one* 2010; **5**: e10153.

77 Wilson GM, Flibotte S, Chopra V, Melnyk BL, Honer WG, Holt RA. DNA copy-number analysis in bipolar disorder and schizophrenia reveals aberrations in genes involved in glutamate signaling. *Human molecular genetics* 2006; **15**: 743–749.

78 Bruckl TM, Uhr M. ABCB1 genotyping in the treatment of depression. *Pharmacogenomics.* 2016; **17**: 2039–2069.

79 Ylikallio E, Woldegebriel R, Tumiati M, Isohanni P, Ryan MM, Stark Z *et al.* MCM3AP in recessive Charcot-Marie-Tooth neuropathy and mild intellectual disability. *Brain a journal of neurology* 2017; **140**: 2093–2103.
